# Routine chest X-rays after pigtail chest tube removal rarely change management in children

**DOI:** 10.1007/s00383-021-04951-w

**Published:** 2021-06-25

**Authors:** Christina M. Theodorou, Mennatalla S. Hegazi, Hope Nicole Moore, Alana L. Beres

**Affiliations:** 1 Department of Pediatric General, Thoracic, and Fetal Surgery, University of California Davis Medical Center, Sacramento, CA USA; 2 Department of Pediatric General, Thoracic, and Fetal Surgery, University of California Davis Medical Center, 2335 Stockton Blvd, Room 5107, Sacramento, CA 95817 USA

**Keywords:** Chest tube, Pigtail thoracostomy, X-ray, Pediatric surgery

## Abstract

**Background:**

The need for chest X-rays (CXR) following large-bore chest tube removal has been questioned; however, the utility of CXRs following removal of small-bore pigtail chest tubes is unknown. We hypothesized that CXRs obtained following removal of pigtail chest tubes would not change management.

**Methods:**

Patients < 18 years old with pigtail chest tubes placed 2014–2019 at a tertiary children’s hospital were reviewed. Exclusion criteria were age < 1 month, death or transfer with a chest tube in place, or pigtail chest tube replacement by large-bore chest tube. The primary outcome was chest tube reinsertion.

**Results:**

111 patients underwent 123 pigtail chest tube insertions; 12 patients had bilateral chest tubes. The median age was 5.8 years old. Indications were pneumothorax (*n* = 53), pleural effusion (*n* = 54), chylothorax (*n* = 6), empyema (*n* = 5), and hemothorax (*n* = 3). Post-pull CXRs were obtained in 121/123 cases (98.4%). The two children without post-pull CXRs did not require chest tube reinsertion. Two patients required chest tube reinsertion (1.6%), both for re-accumulation of their chylothorax.

**Conclusions:**

Post-pull chest X-rays are done nearly universally following pigtail chest tube removal but rarely change management. Providers should obtain post-pull imaging based on symptoms and underlying diagnosis, with higher suspicion for recurrence in children with chylothorax.

## Introduction

Pediatric patients undergo tube thoracostomy for a variety of reasons, including traumatic or spontaneous pneumothorax, hemothorax, pleural effusion or empyema, and following thoracic surgical procedures [1]. Although practice patterns vary by institution, patients routinely have chest X-rays (CXRs) obtained after the removal of the chest tube [2], generally to evaluate for pneumothorax. However, there is mounting evidence that a small pneumothorax in an asymptomatic patient does not always require chest tube reinsertion [3], and that imaging findings from post-pull CXRs rarely change patient management [4, 5]. The majority of these studies have been conducted on patients who have undergone large-bore thoracostomy tube placement.

Pigtail chest tubes are smaller bore (8.5–14 Fr) and are inserted via Seldinger technique, with only a small skin incision necessary. They are being used increasingly in pediatric patients with pathologies including pleural effusion [6] and pneumothorax [7]. Only one study has evaluated the utility of post-pull CXRs in pigtail chest tubes as part of a larger cohort of all pediatric chest tubes, finding only one patient with a pigtail chest tube in whom the post-pull CXR changed management [5]. This study only included 29 patients with pigtail chest tubes, and thus a larger cohort is needed to assess the utility of the post-pull CXR in this population. Additionally, despite mounting evidence that post-pull CXRs are not clinically useful in many cases, they continue to be performed on a routine basis [5].

We hypothesized that CXRs obtained routinely following the removal of a pigtail chest tube in pediatric patients would rarely change patient management. Our primary outcome was chest tube reinsertion following the removal of a pigtail chest tube. Secondary outcomes included chest tube complications.

## Methods

### Study design

All patients less than 18 years old who underwent placement of any chest tube between January 1, 2014 and July 1, 2019 at a tertiary children’s hospital were identified in the electronic medical record using CPT codes, ICD codes, and by querying nursing flowsheets for thoracostomy tubes. Individual chart review was performed to identify patients who had a pigtail chest tube placed. Patients were not included if they underwent large-bore surgical tube thoracostomy or had other pleural drainage devices placed, such as Blake drains placed intraoperatively. Exclusion criteria included age < 1 month at the time of thoracostomy, death or transfer out of the hospital with a chest tube in place, or planned replacement of the pigtail chest tube with a large-bore thoracostomy tube. Data collected included: demographic information, indication for chest tube, chest tube size, laterality, hospital location of placement, role of person placing the chest tube, complications, CXR findings after removal of a pigtail chest tube (post-pull CXR), and unplanned chest tube re-insertions. Chest tube complications were defined as kinking or clogging of the pigtail chest tube, malpositioning, and failure to resolve the underlying reason for insertion (for example, failure to resolve a pleural effusion or pneumothorax). Post-pull CXR results were categorized as stable or worse. Stable was defined as improved, no pneumothorax/effusion, or trace pneumothorax or pleural effusion, or unchanged from previous CXR. Worse was defined as larger pneumothorax or pleural effusion. Institutional Review Board approval was obtained for this study (IRB# 935667) with a waiver of consent.

### Statistical analysis

Descriptive analysis was performed by calculating median and interquartile ranges (IQR) for continuous non-parametric data. Categorical data are presented as number and percentage. Comparisons of categorical data were performed using Fisher’s exact test with significance set at *p* < 0.05. Analysis was conducted using statistical software (GraphPad Prism, version 8).

## Results

### Overall results

There were 111 patients who underwent pigtail chest tube placement between 2014 and 2019. Of these, 12 patients had bilateral pigtail chest tubes, for a total of 123 chest tubes. The median age was 5.8 years old (IQR 2.0–13.4), and 51% were female. The median length of stay (LOS) was 11.7 days (IQR 5.7–31.7).

### Pigtail chest tube placement details

The most common indication for chest tube placement was pleural effusion (*n* = 56, 45.5%), followed by pneumothorax (*n* = 53, 43.1%), chylothorax (*n* = 6, 4.9%), empyema (*n* = 5, 4.1%) and hemothorax (*n* = 3, 2.3%). The different etiologies of pneumothorax were traumatic (*n* = 22 patients), spontaneous (*n* = 12), post-operative (*n* = 9), barotrauma (*n* = 7), iatrogenic during central venous access (*n* = 2), and necrotizing pneumonia (*n* = 1). The majority were inserted by a pediatric intensivist (*n* = 43, 35%), followed by pediatric surgery and trauma surgery (*n* = 18, 14.6% each), and pediatric cardiothoracic surgery and interventional radiology (IR) (*n* = 16, 13% each). The remainder (*n* = 12) were inserted by providers in the emergency department (*n* = 3), neonatal intensive care unit (*n* = 2), adult cardiothoracic surgery (*n* = 1), or at an outside hospital (*n* = 4). In two cases this information was not reported. Pigtail chest tubes were most commonly placed in the pediatric intensive care unit (50.4%) followed by IR (13%), the operating room, and the emergency department (12.2% each). The remainder (*n* = 15) were placed on the pediatric ward (*n* = 4), the surgical intensive care unit (*n* = 2), and the neonatal intensive care unit (*n* = 2). In seven cases, the hospital location of pigtail chest tube placement was not reported. The majority were placed by attending physicians (47%) followed by residents (28%), with 8% inserted by fellows and 6% inserted by nurse practitioners. The role of the inserter was not provided in 14 cases.

There were similar numbers of left and right chest tubes (45% left, 54% right) in patients with unilateral chest tubes. The size of the pigtail chest tubes ranged from 5 to 16 French, with 8.5 French being the most common size (52%). The median chest tube duration was 4.1 days (IQR 2.8–7.5) with one patient having their chest tube in place for 68 days for pleural effusion. Pre-removal chest tube clamp trials were reported in 17/123 cases (13.8%), ranging from 30 min to 5 days in one patient with a complicated history of recurrent pleural effusions, and a total pigtail chest tube duration of 68 days.

### Post-pull CXRs

Post-pull CXRs were done in 121/123 cases (98.4%). These were categorized based on the radiology report as stable (*n* = 112) or worse (*n* = 9). Of the 17 patients who underwent a clamp trial prior to chest tube removal, all post-pull CXRs were reported to be stable. There were 2 chest tube reinsertions, both in children with chylothorax: one had a stable post-pull CXR but the chylothorax reaccumulated over the following four days resulting in chest tube re-insertion. The other patient had a worse post-pull CXR reading and had a chest tube reinserted 2 days later for continued re-accumulation of the chylothorax. Thus, no children had immediate interventions as a result of the post-pull CXR alone.

Only two patients did not have a post-pull CXR performed. Both of the patients without post-pull CXRs were observed for 4 h following chest tube removal and discharged home. One patient with apical blebs had a scheduled follow-up CXR the next week prior to planned video-assisted thoracoscopic surgery (VATS) with blebectomy. The CXR revealed a moderate pneumothorax, but the patient was asymptomatic and was readmitted for his scheduled VATS as planned; the chest tube was not reinserted prior to surgery.

### Complications

There were 10 pigtail chest tube-related complications (8.1%). Four tubes were found to be kinked, of which 1 required manipulation at the bedside; all of these tubes were 8.5 Fr. Two were accidentally pulled back, with 1 outside the thoracic cavity, however, tube replacement was not needed. Two clogged, with one requiring thrombolytic therapy (8.5 Fr) and one requiring tube replacement (5 Fr). Two chest tubes had to be removed and replaced due to failure to resolve the underlying pleural effusion (14 Fr) or pneumothorax (8.5 Fr). When analyzed by the size of chest tube, 11.0% of pigtail chest tubes sized 8.5 Fr or smaller had a complication compared to only one complication among patients with a pigtail chest tube larger than 8.5 Fr (3.1%, *p* = 0.3).

There were 13 ED visits within 30 days (11.8%) and 11 readmissions (10%). One patient was readmitted 12 days after discharge with shortness of breath and was found to have underlying mediastinal lymphoma and associated pleural effusion, which required pigtail chest tube replacement 15 days after the prior tube has been removed. The other ED visits and readmissions were for reasons unrelated to the removal of the pigtail chest tube. One patient died before discharge after being transitioned to comfort care for anoxic brain injury, over a month after the pigtail chest tube was removed.

## Discussion

In this study, the largest study of pediatric patients undergoing pigtail chest tube placement to date to the best of our knowledge in the English literature, we found that post-pull CXRs are obtained in almost all children, however these images very rarely change patient management, and none resulted in immediate intervention. Reinsertion of the chest tube was only required in two cases, both more than 24 h after removal of the chest tube for worsening fluid accumulation in the chest. Even in these two cases, therefore, it was not the initial CXR, but subsequent follow-up CXRs which prompted an intervention. Both of these children had chylothorax. Only two patients did not have post-pull CXRs and neither required chest tube replacement. Complications such as kinking or clogging of chest tubes were more common in smaller bore (8.5 Fr or smaller) pigtail chest tubes.

Pigtail chest tubes have been found to be an effective treatment in pediatric parapneumonic effusion [6], empyema [8], and pneumothorax [7, 9, 10]. There is data suggesting 14 Fr pigtail chest tubes are not inferior to larger bore (28–40 Fr) chest tubes in adult patients with hemothorax and in animal models [11–13]. In our cohort, there were more complications observed in patients with 8.5 Fr or smaller pigtail chest tubes, however the overall complication rate was very low at 8.1%.

Pigtail chest tubes are a smaller bore and patients report less pain at the insertion site when compared to large-bore chest tubes [14]. During removal of large-bore chest tubes, it is possible for air to enter the thoracic cavity and cause a pneumothorax which may be evident on the post-pull CXR. However, multiple studies of large-bore chest tubes have examined the utility of post-pull CXRs in both pediatric [4, 5, 15–17], and adult patients [3, 18–20] and found that post-pull CXRs only rarely change patient management, and in most cases, additional interventions are predicted by clinical symptoms and signs. Post-pull CXRs in neonates, who are less able to express symptoms such as shortness of breath, have also been found to have low utility, and rarely predict the need for chest tube reinsertion [21]. Similar results have been seen in studies of mechanically ventilated patients [22].

As pigtail chest tubes are inserted via Seldinger technique, there is a smaller incision just in the skin on the chest wall, and a theoretically lower risk of air entrapment at the time of chest tube removal. In a previous smaller study of post-pull CXRs, pigtail chest tubes had only 3.4% replacement rate compared to 6% for large-bore chest tubes [5]. The current study is the largest reported cohort of pediatric patients with pigtail chest tubes placed, and our results confirm those of previous studies of patients with large-bore chest tubes: both recurrence of the underlying pathology and chest tube reinsertion are rare events. Post-pull CXRs for pediatric patients with pigtail chest tubes have a very low utility for most patients. The only two patients who required chest tube reinsertion in our study both had chylothorax, and post-pull CXRs may be warranted in this population; however, neither patient required any immediate intervention. It is of note that our study only included six patients with chylothorax so larger studies of that population are needed. Additionally, the utility of chest tube clamp trials prior to removal for pigtail catheters is currently unknown and requires further study.

### Limitations

This study has several limitations. It is a retrospective chart review of a single institution, and results may differ at other institutions. Some patients who had pigtail chest tubes placed may have been missed due to coding inaccuracies. The majority of patients had pneumothorax or pleural effusion, with low rates of empyema, hemothorax, and chylothorax, so our results may not be generalizable to all patients with these conditions. We did not evaluate neonates, who represent a different population with differing physiology and underlying pathology and our results should not be applied to this population without further study.

## Conclusion

Post-pull CXRs are done on almost every pediatric patient following removal of a pigtail chest tube, but the findings rarely result in a change in management. Reinsertion of the chest tube is rare, occurring in only 1.6% of cases, and no patient underwent immediate intervention based on the result of the post-pull CXR. Imaging following chest tube removal may not be necessary in the majority of patients in the absence of clinical indications; however, patients with chylothorax had a higher rate of chest tube reinsertion and warrant a lower threshold for obtaining follow up imaging.

## References

[1] Strutt J, Kharbanda A (2015). Pediatric chest tubes and pigtails: an evidence-based approach to the management of pleural space diseases. Pediatr Emerg Med Pr.

[2] Martino K, Merrit S, Boyakye K, Sernas T, Koller C, Hauser CJ (1999). Prospective randomized trial of thoracostomy removal algorithms. J Trauma Inj Infect Crit Care.

[3] Palesty JA, McKelvey AA, Dudrick SJ (2000). The efficacy of x-rays after chest tube removal. Am J Surg.

[4] Cunningham JP, Knott EM, Gasior AC, Juang D, Snyder CL, St. Peter SD (2014). Is routine chest radiograph necessary after chest tube removal?. J Pediatr Surg.

[5] Johnson B, Rylander M, Beres AL (2017). Do X-rays after chest tube removal change patient management?. J Pediatr Surg.

[6] Lin CH, Lin WC, Chang JS (2011). Comparison of pigtail catheter with chest tube for drainage of parapneumonic effusion in children. Pediatr Neonatol.

[7] Kuo HC, Lin YJ, Huang CF, Chien SJ, Lin IC, Lo MH (2013). Small-bore pigtail catheters for the treatment of primary spontaneous pneumothorax in young adolescents. Emerg Med J.

[8] Petel D, Li P, Emil S (2013). Percutaneous pigtail catheter versus tube thoracostomy for pediatric empyema: a comparison of outcomes. Surg (United States).

[9] Dull KE, Fleisher GR (2002). Pigtail catheters versus large-bore chest tubes for pneumothoraces in children treated in the emergency department. Pediatr Emerg Care.

[10] Wei YH, Lee CH, Cheng HN, Ten TL, Hsiao CC (2014). Pigtail catheters versus traditional chest tubes for pneumothoraces in premature infants treated in a neonatal intensive care unit. Pediatr Neonatol.

[11] Kulvatunyou N, Joseph B, Friese RS, Green D, Gries L, O’Keeffe T (2012). 14 French pigtail catheters placed by surgeons to drain blood on trauma patients: Is 14-Fr too small?. J Trauma Acute Care Surg.

[12] Bauman ZM, Kulvatunyou N, Joseph B, Jain A, Friese RS, Gries L (2018). A prospective study of 7-year experience using percutaneous 14-french pigtail catheters for traumatic hemothorax/hemopneumothorax at a level-1 trauma center: size still does not matter. World J Surg.

[13] Russo RM, Zakaluzny SA, Neff LP, Grayson JK, Hight RA, Galante JM (2015). A pilot study of chest tube versus pigtail catheter drainage of acute hemothorax in swine. J Trauma Acute Care Surg.

[14] Kulvatunyou N, Erickson L, Vijayasekaran A, Gries L, Joseph B, Friese RF (2014). Randomized clinical trial of pigtail catheter versus chest tube in injured patients with uncomplicated traumatic pneumothorax. Br J Surg.

[15] McGrath E, Ranstrom L, Lajoie D, McGlynn L, Mooney D (2017). Is a chest radiograph required after removal of chest tubes in children?. J Pediatr Heal Care.

[16] Pacharn P, Heller DND, Kamnen BF, Bryce TJ, Reddy MV, Bailey RA (2002). Are chest radiographs routinely necessary following thoracostomy tube removal?. Pediatr Radiol.

[17] Woodward CS, Dowling D, Taylor RP, Savin C (2013). The routine use of chest radiographs after chest tube removal in children who have had cardiac surgery. J Pediatr Heal Care.

[18] McCormick JT, O’Mara MS, Papasavas PK, Caushaj PF (2002). The use of routine chest x-ray films after chest tube removal in postoperative cardiac patients. Ann Thorac Surg.

[19] Goodman MD, Huber NL, Johannigman JA, Pritts TA (2010). Omission of routine chest x-ray after chest tube removal is safe in selected trauma patients. Am J Surg.

[20] Pacanowski JP, Waack ML, Daley BJ, Hunter KS, Clinton R, Diamond DL (2000). Is routine roentgenography needed after closed tube thoracostomy removal?. J Trauma Inj Infect Crit Care.

[21] Van Den Boom J, Battin M (2007). Chest radiographs after removal of chest drains in neonates: clinical benefit or common practice?. Arch Dis Child Fetal Neonatal Ed.

[22] Pizano LR, Houghton DE, Cohn SM, Frisch MS, Grogan RH (2002). When should a chest radiograph be obtained after chest tube removal in mechanically ventilated patients? A prospective study. J Trauma Inj Infect Crit Care.

